# Long-term visit-to-visit blood pressure variability and risk of cardiovascular and bleeding events: insights from the ENGAGE AF-TIMI 48 trial

**DOI:** 10.1038/s41440-024-02083-x

**Published:** 2025-02-19

**Authors:** Riccardo Proietti, Michael Gerard Palazzolo, Christian T. Ruff, Gregory Y. H. Lip, Robert P. Giugliano

**Affiliations:** 1 Liverpool Centre for Cardiovascular Science at University of Liverpool, Liverpool John Moores University and Liverpool Heart & Chest Hospital, Liverpool, UK; 2 TIMI Study Group, Brigham and Women’s Hospital and Harvard Medical School, Boston, MA USA

**Keywords:** blood pressure variability, atrial fibrillation, bleeding, cardiovascular outcomes

## Abstract

This post hoc analysis of the ENGAGE AF-TIMI 48 trial assesses differences in cardiovascular and bleeding events according to visit-to-visit blood pressure variability (BPv) in 19,680 patients with a minimum of 4 blood pressure measurements post randomization. Patients were categorized into four groups based on the standard deviation of systolic blood pressure (SBP-SD). In comparisons of the fourth vs first quartile of SBP-SD adjusted for components of the CHA_2_D_2_-VASc score and baseline SBP, there were no differences in the odds of stroke, cardiovascular mortality, or all-cause mortality. However, there were statistically significant increases in the risk of major bleeding (OR 1.9, (1.6–2.25)). myocardial infarction (OR 1.42 (1.08–1.87)) and heart failure outcomes (OR 1.49 (1.3–1.72)) in the fourth quartile of BPv. This post-hoc analysis shows that BPv is independently associated with an increased risk of bleeding, MI, and heart failure outcomes in a population with AF on oral anticoagulation.

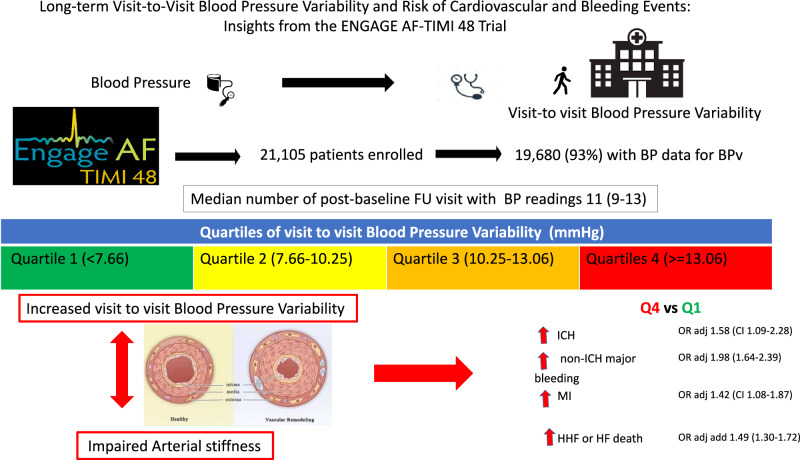

## Introduction

Prior studies demonstrate that ambulatory visit-to-visit blood pressure variability (BPv) was an independent risk factor for cardiovascular events [1]. In patients with hypertension and high cardiovascular risk, greater BPv, has been associated with a higher risk for stroke, myocardial infarction, heart failure, and mortality [1–3]. In patients with atrial fibrillation (AF), BPv was an independent predictor of stroke, major bleeding and quality of anticoagulation control [4, 5].

The aim of this post hoc analysis of the Effective Anticoagulation with Factor Xa Next Generation in Atrial Fibrillation–Thrombolysis in Myocardial Infarction 48 trial (ENGAGE AF-TIMI 48) [6] was to investigate the relationship between BPv and cardiovascular and bleeding events in patients with AF.

## Methods

ENGAGE AF-TIMI 48 is a multinational, multicentre double-blind, randomized trial comparing 2 dose regimens of edoxaban with warfarin in patients with AF. The study protocol and the principal results have been described in detail elsewhere [6]. BP measurement was performed at the baseline visit and at every follow-up visit, according to the protocol defined schedule. Systolic (SBP) and diastolic BP (DBP) measurements were performed by the investigator or designee while the patient was seated after >5 minutes of rest. If a subject had multiple measurements in a single visit the measurements were averaged. SBP visit-to-visit variability was defined according to the standard deviation (SD) of mean SBP during follow-up visits for every patient, as for the recommendation of the European Society of Hypertension [7]. Patients were categorized according to the quartiles of the SD of their SBP. Additionally, the Coefficient of Variation (CoV) for BPv, defined as the ratio of SD to the mean BP, was calculated. A sensitivity analysis was conducted using both quartiles and the continuous variable of CoV to evaluate potential bias in the correlation between SD and average BPv.Thromboembolic risk was defined according to the CHA_2_DS_2_-VASc risk score. Haemorrhagic risk was calculated according to HAS-BLED score. However, the “L” criteria could not be assessed in the ENGAGE AF-TIMI 48 as 60% were not previously on vitamin K antagonist (VKA).

Outcomes of interest for this sub analysis were (1) all stroke, as well as ischaemic and haemorrhagic stroke separately, (2) major bleeding, as well as intracranial haemorrhage (ICH) and major non-ICH bleeding separately, (3) myocardial infarction (MI), (4) composite of heart failure hospitalization (HHF) and death due to heart failure (HF), (5) cardiovascular death; (6) all-cause mortality; and (7) the net outcome consisting of stroke, major bleeding, and cardiovascular death.

All continuous variables were expressed as mean and SD or median and interquartile range (IQR) and compared accordingly with 1-way ANOVA test.

A logistic regression model, adjusted for the individual components of the CHA_2_DS_2_-VASc score and baseline systolic blood pressure, was constructed to establish the relationship of continuous SBP-SD with visit-to-visit BPv.

## Results

Of the 21,105 patients enroled in the ENGAGE AF-TIMI 48 Trial, 19,680 (93%) had sufficient BP data for the current analysis. The median (IQR) follow-up was 1022 (891–1170) days, age 72 (64–77) years, CHA_2_DS_2_-VASc score 4 (3–5) and HAS-BLED score 2 (2–3). There were 7426 (37.7%) women and 10191 (51.8%) patients had permanent AF. The median number of post-baseline BP readings was 11 (9–13). During follow-up, the mean (SD) baseline SBP was 130.17 (15.24)). Baseline characteristics varied significantly according to SBP-SD quartiles (1st, <7.66; 2nd, 7.66–10.25; 3rd, 10.25–13.06; and 4th, ≥13.06 mm Hg) (Table 1). In general, patients with higher BPv were at higher risk. There were no statistically significant differences in baseline characteristics within SBP-SD quartile by randomized treatment group.

**Table 1:** Baseline Characteristic of Patients by Quartiles of Systolic Blood Pressure Variability

	Overall	Q1 ( < 7.66) (*N* = 4920)	Q2 (7.66- < 10.25) (*N* = 4923)	Q3 (10.25- < 13.06) (*N* = 4917)	Q4 ( > = 13.06) (*N* = 4920)	*P*-trend
Age (years) - Median (IQR)	72 (64–77)	70 (62–76)	71 (63–77)	72 (65–78)	74 (67–79)	<0.001
Female sex	7426 (37.7%)	1762 (35.8%)	1823 (37%)	1833 (37.3%)	2008 (40.8%)	<0.001
Region						
North America	4321 (22%)	786 (16%)	1024 (20.8%)	1172 (23.8%)	1339 (27.2%)	<0.001
Latin America	2455 (12.5%)	490 (10%)	594 (12.1%)	624 (12.7%)	747 (15.2%)	<0.001
Western Europe	2970 (15.1%)	521 (10.6%)	709 (14.4%)	818 (16.6%)	922 (18.7%)	<0.001
Eastern Europe	6788 (34.5%)	2503 (50.9%)	1797 (36.5%)	1418 (28.8%)	1070 (21.7%)	<0.001
Asia	3146 (16%)	620 (12.6%)	799 (16.2%)	885 (18%)	842 (17.1%)	<0.001
Body mass index (kg/m2) – Median (IQR)	28.7 (25.5–32.7)	28.8 (25.7–32.7)	28.7 (25.5–32.6)	28.7 (25.4–32.6)	28.4 (25.3–32.7)	0.038
Type of atrial fibrillation						
Paroxysmal	4975 (25.3%)	1149 (23.4%)	1195 (24.3%)	1212 (24.7%)	1419 (28.8%)	<0.001
Persistent	4510 (22.9%)	1109 (22.5%)	1130 (23%)	1154 (23.5%)	1117 (22.7%)	<0.001
Permanent	10191 (51.8%)	2662 (54.1%)	2597 (52.8%)	2549 (51.9%)	2383 (48.4%)	<0.001
Comorbidities						
Coronary artery disease	6507 (33.1%)	1759 (35.8%)	1589 (32.3%)	1569 (31.9%)	1590 (32.3%)	<0.001
Congestive heart failure	11262 (57.2%)	3221 (65.5%)	2857 (58%)	2634 (53.6%)	2550 (51.8%)	<0.001
Peripheral Arterial Disease	785 (4%)	186 (3.8%)	157 (3.2%)	205 (4.2%)	237 (4.8%)	0.001
Diabetes mellitus	7135 (36.3%)	1619 (32.9%)	1764 (35.8%)	1843 (37.5%)	1909 (38.8%)	<0.001
Dyslipidemia	10415 (52.9%)	2445 (49.7%)	2547 (51.7%)	2691 (54.7%)	2732 (55.5%)	<0.001
Hypertension	18435 (93.7%)	4611 (93.7%)	4551 (92.4%)	4607 (93.7%)	4666 (94.8%)	0.003
Stroke/Triansient Ischemic Attack	5557 (28.2%)	1346 (27.4%)	1369 (27.8%)	1418 (28.8%)	1424 (28.9%)	0.044
Charlson Comorbidity Index (CCI)						
Median (IQR)	3 (2–4)	3 (2–4)	3 (2–3)	3 (2–4)	3 (2–4)	<0.001
Mean (SD)	2.9 (1.1)	2.9 (1.1)	2.8 (1.1)	2.9 (1.1)	3 (1.2)	<0.001
0–2	8013 (40.7%)	2156 (43.8%)	2144 (43.6%)	1943 (39.5%)	1770 (36%)	<0.001
>=3	11667 (59.3%)	2764 (56.2%)	2779 (56.4%)	2974 (60.5%)	3150 (64%)	<0.001
CHA_2_DS_2_–VASc score						
Median (IQR)	4 (3–5)	4 (3–5)	4 (3–5)	4 (3–5)	4 (4–5)	<0.001
Mean (SD)	4.3 (1.4)	4.2 (1.4)	4.2 (1.4)	4.3 (1.4)	4.5 (1.4)	<0.001
0–3	5864 (29.8%)	1677 (34.1%)	1575 (32%)	1426 (29%)	1186 (24.1%)	<0.001
>=4	13816 (70.2%)	3243 (65.9%)	3348 (68%)	3491 (71%)	3734 (75.9%)	<0.001
HAS–BLED Score						
Median (IQR)	2 (2–3)	2 (2–3)	2 (2–3)	2 (2–3)	3 (2–3)	<0.001
Mean (SD)	2.5 (1)	2.4 (1)	2.4 (1)	2.5 (1)	2.6 (1)	<0.001
0–2	10643 (54.1%)	2899 (58.9%)	2772 (56.3%)	2567 (52.2%)	2405 (48.9%)	<0.001
>=3	9037 (45.9%)	2021 (41.1%)	2151 (43.7%)	2350 (47.8%)	2515 (51.1%)	<0.001
Systolic Blood Pressure						
Total number of post-baseline records - Median (IQR)	11 (9–13)	11 (8–13)	12 (10–14)	12 (10–14)	11 (9–13)	<0.001
Baseline (mmHg) - Median (IQR)	130 (120–140)	130 (120–140)	130 (120–140)	130 (120–140)	132 (120–144)	<0.001
SD (mmHg) - Median (IQR)	10.2 (7.7–13.1)	6 (4.9–7)	9 (8.4–9.6)	11.5 (10.8–12.3)	15.4 (14.1–17.7)	<0.001
CoV (%) - Median (IQR)	8.1 (6.1–10.2)	4.7 (3.8–5.5)	7.1 (6.5–7.7)	9.1 (8.4–9.8)	12 (10.9–13.6)	<0.001
Diastolic BP						
Total number of post-baseline records - Median (IQR)	11 (9–13)	11 (8–13)	12 (10–14)	12 (10–14)	11 (9–13)	<0.001
Baseline (mmHg) - Median (IQR)	80 (70–85)	80 (71–84)	80 (70–83)	80 (70–85)	80 (70–85)	<0.001
SD (mmHg) - Median (IQR)	7 (5.3–8.7)	5.3 (4–6.8)	6.5 (5.2–7.9)	7.4 (6–8.9)	8.7 (7.1–10.6)	<0.001
CoV (%) - Median (IQR)	9.1 (6.9–11.6)	6.8 (5.1–8.9)	8.6 (6.8–10.6)	9.7 (7.8–11.8)	11.4 (9.3–14)	<0.001
Heart rate (bmp) - Median (IQR)	72 (64–82)	74 (65–83)	72 (64–82)	72 (64–82)	72 (64–81)	<0.001
Creatinine clearance (mL/min) - Median (IQR)	70.9 (54.3–92.5)	75.2 (57.3–98.3)	72.7 (56.4–94.1)	69.7 (53.8–91)	66.3 (50.9–86.2)	<0.001
Haemoglobin (g/dL) - Median (IQR)	14.1 (13.1–15)	14.2 (13.2–15.1)	14.1 (13.1–15.1)	14 (13.1–15)	13.9 (12.9–14.9)	<0.001
Time-in-Therapeutic Range - Median (IQR)	69 (58.1–77.5)	67.1 (54–77.1)	70.3 (59.9–78.7)	70.1 (60.8–77.9)	68.1 (57.2–76.5)	0.260
Medications						
Any antiplatelet (aspirin, thienopyridine, or other)	6117 (31.1%)	1536 (31.2%)	1453 (29.5%)	1533 (31.2%)	1595 (32.4%)	0.072
Aspirin	5663 (28.8%)	1435 (29.2%)	1355 (27.5%)	1414 (28.8%)	1459 (29.7%)	0.344
Others (anti-platelet other than ASA or thienopyridine)	157 (0.8%)	35 (0.7%)	38 (0.8%)	40 (0.8%)	44 (0.9%)	0.298
Beta-blockers	13069 (66.4%)	3394 (69%)	3270 (66.4%)	3200 (65.1%)	3205 (65.1%)	<0.001
Calcium channel blockers	4537 (23.1%)	1081 (22%)	1122 (22.8%)	1155 (23.5%)	1179 (24%)	0.013
Statin	3991 (20.3%)	772 (15.7%)	929 (18.9%)	1076 (21.9%)	1214 (24.7%)	<0.001
Randomization Group						
Warfarin	6570 (33.4%)	1623 (33%)	1690 (34.3%)	1591 (32.4%)	1666 (33.9%)	0.449
Lower-dose edoxaban regimen	6580 (33.4%)	1655 (33.6%)	1610 (32.7%)	1664 (33.8%)	1651 (33.6%)	0.449
Higher-dose edoxaban regimen	6530 (33.2%)	1642 (33.4%)	1623 (33%)	1662 (33.8%)	1603 (32.6%)	0.449

No statistically significant differences were present between Q4 and Q1 of SBP-SD in the adjusted odds for stroke (OR 1.03, CI 0.86–1.24), cardiovascular death (OR 0.89, CI 0.77–1.04) or all-cause mortality (0.93, CI 0.82–1.06) (Table 2). However, there were statistically significant higher risks of major bleeding (OR 1.9, 1.6–2.25)), including both ICH (OR 1.58, CI 1.09–2.28) and non-ICH major bleeding (OR 1.98 (1.64–2.39)); MI (OR 1.42, CI 1.08–1.87); HHF or HF death (OR add 1.49 (1.3–1.72); and the net outcome (OR 1.28, CI 1.15–1.43) in Q4 vs Q1 of BPv. The sensitivity analysis repeated for quartiles of CoV showed similar results (supplementary table 1).

**Table 2:** Clinical Outcomes Across Quartiles of Standard Deviation of Systolic Blood Pressure Variability and by Continuous Standard Deviation of Systolic Blood Pressure Variability (Adjusted Model^a^)

	Quartile 1 ( < 7.66)			Quartile 2 (7.66–10.25)			Quartile 3 (10.25–13.06)			Quartile 4 ( > = 13.06)			Continuous variable Adj OR (95% CI) per SD of BPv/ p-value
Outcome	n/N	Rate (%yr)	Adj OR (95% CI)	*n*/*N*	Rate (%/yr)	Adj OR (95% CI)	n/N	Rate (%/yr)	Adj OR (95% CI)	*n*/*N*	Rate (%/yr)	Adj OR (95% CI)	
**Stroke**	232/4920	1.78	Ref	163/4923	1.19	0.68 (0.55–0.83)	170/4917	1.23	0.68 (0.55–0.83)	267/4920	2.00	1.03 (0.86–1.24)	1.08 (1.01–1.16)/0.019
**Ischaemic**	198/4920	1.51	Ref	140/4923	1.02	0.69 (0.55–0.86)	144/4917	1.04	0.68 (0.55–0.85)	224/4920	1.67	1.01 (0.83–1.24)	1.07(0.99–1.15)/0.080
**Haemorrhagic**	36/4920	0.27	Ref	24/4923	0.17	0.64 (0.38–1.08)	27/4917	0.19	0.67 (0.4–1.11)	52/4920	0.38	1.26 (0.82–1.95)	1.2(1.04–1.38)/0.012
**Major Bleeding**	219/4915	1.98	Ref	245/4920	1.97	1.11 (0.92–1.33)	315/4912	2.57	1.39 (1.16–1.66)	437/4918	3.92	1.9 (1.6–2.25)	1.29(1.23–1.36)/ < 0.001
**ICH**	46/4915	0.41	Ref	32/4920	0.25	0.67 (0.42–1.05)	44/4912	0.35	0.88 (0.58–1.34)	82/4918	0.71	1.58 (1.09–2.28)	1.3(1.17–1.45)/ < 0.001
**Non-ICH**	173/4915	1.56	Ref	216/4920	1.74	1.25 (1.01–1.53)	274/4912	2.24	1.54 (1.26–1.87)	359/4918	3.22	1.98 (1.64–2.39)	1.28(1.21–1.35)/ < 0.001
**Myocardial Infarction**	89/4920	0.67	Ref	70/4923	0.51	0.78 (0.57–1.07)	98/4917	0.71	1.03 (0.77–1.38)	140/4920	1.04	1.42 (1.08–1.87)	1.2(1.1–1.3)/ < 0.001
**HHF or HF death**	382/4920	2.96	Ref	373/4923	2.77	1.01 (0.87–1.17)	463/4917	3.47	1.3 (1.13–1.5)	523/4920	4.03	1.49 (1.3–1.72)	1.23(1.17–1.29)/ < 0.001
**CV Death**	393/4920	2.93	Ref	238/4923	1.71	0.59 (0.5–0.7)	249/4917	1.78	0.59 (0.5–0.7)	367/4920	2.67	0.89 (0.77–1.04)	1.03(0.97–1.09)/0.299
**All-cause Death**	543/4920	4.05	Ref	337/4923	2.42	0.58 (0.5–0.67)	366/4917	2.61	0.61 (0.53–0.7)	544/4920	3.95	0.93 (0.82–1.06)	1.04(0.99–1.09)/0.117
**Net Outcome**^b^	732/4920	5.74	Ref	588/4923	4.39	0.77 (0.68–0.86)	666/4917	5.00	0.85 (0.75–0.95)	963/4920	7.58	1.28 (1.15–1.43)	1.17(1.13–1.22)/ < 0.001

^a^Model adjusted for baseline SBP, congestive heart failure, hypertension, age, diabetes mellitus, stroke/transient ischaemic attack, vascular disease including either myocardial infarction or peripheral arterial disease, and sex)

^b^Net outcome: Stroke, major bleeding, cardiovascular death

*CV* cardiovascular, *HF* heart failure, *HHF* hospitalized for heart failure, *ICH* intracranial haemorrhage, *SBP* systolic blood pressure

Considering visit-to-visit BPv as a continuous variable, generally similar findings were found as in the quartile analysis. The analysis BPv as a continuous variable revealed increased risks of higher BPv and the risks of haemorrhagic stroke (OR 1.2 95% CI (1.04–1.38) but not ischaemic stroke (OR 1.07, 95% CI (0.99–1.15).

There was an increase in the odds of major bleeding for every SD increase in visit-to-visit BPv (OR 1.29, CI 1.23–1.36), including both ICH OR 1.3 (1.17–1.45), and non-ICH major bleeding (1.28 (1.21–1.35)) separately. For every increase in SD in visit-to-visit BPv an increase in the odds of MI (OR 1.20, CI 1.10–1.3), HHF or death (OR 1.23, CI 1.17–1.29) and net outcome (OR 1.17, CI 1.13–1.22).

In the analysis conducted for CoV as a continuous variables a significant association between CV and all cause of death was detected (supplementary table 1), which was not significant in SD BPv analysis as continuous variables.

Across quartiles of visit-to-visit SBP-SD, there was no evidence of significant effect modification by BPv on the relationships between randomized anticoagulant treatment groups and outcomes.

## Conclusion

The main findings of this post-hoc analysis of the relationship between visit-to-visit BPv and outcomes from the ENGAGE AF-TIMI 48 trial showed that higher BPv were: (i) associated with increased adjusted risks of major bleeding (including both ICH and non-ICH major bleeding separately) and major cardiovascular events including, MI, HHF or HF death, and net outcomes; (ii) strongly associated haemorrhagic stroke, but not ischaemic stroke; (iii) not associated with CV or total mortality. The distribution of BPv was similar in the warfarin and edoxaban arms with no evidence of effect modification by BPv on the relationships between anticoagulants and outcomes.

Mechanistically BPv has been linked to vascular stiffness; our finding suggests that the biological significance of BPv may be rooted in a vascular wall remodelling, which increases susceptibility to bleeding. Small vessels disease of cerebral arteries is considered a marker of organ damage related to increased pulsatile blood pressure which is the clinical outcome of arterial stiffens [8]. In addition, arterial stiffness has been associated with an increased risk of cerebral microbleeds and enlarged perivascular spaces [9] which have been amply associated with risk of ICH. Compared to cerebral microbleeds and enlarged perivascular space that require CT scan, BPv can be easily and harmlessly assessed in a clinical setting. Intracranial bleeding is one of the most severe and unpredictable clinical events and in patients with AF the occurrence of intracranial bleeding offsets the benefit of anticoagulation therapy.

Of note, our finding are aligned with a recent sub-analysis of the ASCOT trial [10] that suggests a SD systolic BPv cut-off of 13 as an increased risk for major cardiovascular events independently from controlled BP. Indeed, in the quartile-based analysis the Q4 (≥13.06 mm Hg) was associated with an increased odd for major CV (MI, HHF and HF death, net outcome).

A positive association was observed between BPv and both CV and total mortality only when CoV was analysed as a continuous variable. In contrast to prior reports, our analysis by quartiles did not show a significant association for both SD and CoV.Various factors should be considered when assessing this outcome: (1) differences in study design (randomized controlled trial versus registry) [4], (2) variation in primary therapy (anti-coagulation therapies versus rhythm control) [5], (3) population differences (prevalence of heart failure 58% versus 21%) [5, 6]. Of note, mortality in the ENGAGE AF-TIMI 48 trial was predominantly cardiovascular (71%), with 45% attributed to sudden cardiac death which can explain the lack of association we found in our analysis with BPv [11].

The absence of the effect of oral anticoagulation treatment suggests established vascular remodelling that is difficult to reverse pharmacologically, additionally, the study’s follow-up may be too short for such drug-induced remodelling.

An important limitation of our study was that standardized instructions on BP measurements with repeat assessments to minimize measurement error were not mandated in ENGAGE AF-TIMI 48 [6]. For some outcomes (e.g., stroke) the event rates were J-shaped across the range of SBP-SD. However, the magnitude of the differences in the lower quartiles was small and data do not support a significant difference (maybe play of chance). On the other hand, at the 4th quartile there were significantly higher risks of several events.

Our results harbour clinical relevance pointing out BPv as a possible biomarker for risk stratification in populations at higher risk, particularly capturing the risk of bleeding.

## Supplementary information

Supplemental Table 1

## Data Availability

The full database for this trial cannot be shared, but researchers interested in collaboration should contact the corresponding author.
