# Supplementary material for: Long-term visit-to-visit blood pressure variability and risk of cardiovascular and bleeding events: insights from the ENGAGE AF-TIMI 48 trial
Source: Hypertens Res. 2025 Feb 19;48(4):1613–8. doi: 10.1038/s41440-024-02083-x (PMC11972953; doi:10.1038/s41440-024-02083-x)
Supplement: Supplementary file 1 — Supplemental Table 1 [file 41440_2024_2083_MOESM1_ESM.docx]

| **Supplemental Table 1 Clinical Outcomes Across Quartiles of Coefficient of Variance of Systolic Blood Pressure Variability and by Continuous Coefficient of Variance of Systolic Blood Pressure Variability (Adjusted Model*)** | | | | | | | | | | | | | |
| --- | --- | --- | --- | --- | --- | --- | --- | --- | --- | --- | --- | --- | --- |
|  | **Quartile 1 (<6.06)** | | | **Quartile 2(6.06-8.06)** | | | **Quartile 3 (8.06-10.23)** | | | **Quartile 4 (>=10.23)** | | | **Continuous variable**  **Adj OR (95% CI) per CoV of BPv/ p-value** |
| **Outcome** | **n/N** | **Rate (%yr)** | **Adj OR (95% CI)** | **n/N** | **Rate (%/yr)** | **Adj OR (95% CI)** | **n/N** | **Rate (%/yr)** | **Adj OR (95% CI)** | **n/N** | **Rate (%/yr)** | **Adj OR (95% CI)** |  |
| **Stroke** | **234/4920** | **1.79** | **Ref** | **168/4920** | **1.23** | **0.68 (0.56-0.84)** | **167/4920** | **1.21** | **0.68 (0.55-0.83)** | **263/4920** | **1.98** | **1.04 (0.87-1.25)** | **1.07 (1-1.14)/0.066** |
| **Ischemic** | **200/4920** | **1.52** | **Ref** | **147/4920** | **1.07** | **0.71 (0.57-0.88)** | **144/4920** | **1.04** | **0.69 (0.55-0.86)** | **215/4920** | **1.67** | **0.99 (0.81-1.21)** | **1.05 (0.98-1.13)/0.171** |
| **Haemorrhagic** | **36/4920** | **0.27** | **Ref** | **23/4920** | **0.17** | **0.58 (0.34-0.99)** | **24/4920** | **0.17** | **0.63 (0.37-1.06)** | **56/4920** | **0.41** | **1.43 (0.93-2.19)** | **1.18 (1.02-1.38)/0.030** |
| **Major Bleeding** | **215/4915** | **1.93** | **Ref** | **248/4916** | **2.02** | **1.14 (0.94-1.37)** | **307/4917** | **2.49** | **1.37 (1.15-1.64)** | **446/4917** | **4.02** | **1.99 (1.68-2.36)** | **1.32 (1.25-1.39)/<0.001** |
| **ICH** | **41/4915** | **0.36** | **Ref** | **35/4916** | **0.28** | **0.81 (0.51-1.28)** | **45/4917** | **0.36** | **1.02 (0.67-1.57)** | **83/4917** | **0.73** | **1.82 (1.24-2.66)** | **1.31 (1.16-1.47)/<0.001** |
| **Non-ICH** | **174/4915** | **1.56** | **Ref** | **215/4916** | **1.75** | **1.23 (1-1.51)** | **266/4917** | **2.15** | **1.48 (1.21-1.8)** | **367/4917** | **3.30** | **2.02 (1.67-2.43)** | **1.31 (1.24-1.39)/<0.001** |
| **Myocardial Infarction** | **88/4920** | **0.66** | **Ref** | **63/4920** | **0.46** | **0.7 (0.51-0.98)** | **107/4920** | **0.77** | **1.14 (0.85-1.52)** | **139/4920** | **1.04** | **1.44 (1.09-1.89)** | **1.22 (1.12-1.34)/<0.001** |
| **HHF or HF death** | **360/4920** | **2.78** | **Ref** | **346/4920** | **2.58** | **1.01 (0.87-1.19)** | **453/4920** | **3.37** | **1.32 (1.14-1.53)** | **582/4920** | **4.53** | **1.74 (1.51-2)** | **1.28 (1.22-1.34)/<0.001** |
| **CV Death** | **375/4920** | **2.79** | **Ref** | **234/4920** | **1.68** | **0.61 (0.52-0.72)** | **241/4920** | **1.71** | **0.59 (0.5-0.7)** | **397/4920** | **2.90** | **0.99 (0.85-1.16)** | **1.06 (1-1.13)/0.033** |
| **All-cause Death** | **519/4920** | **3.86** | **Ref** | **334/4920** | **2.40** | **0.6 (0.52-0.7)** | **344/4920** | **2.45** | **0.59 (0.51-0.68)** | **593/4920** | **4.33** | **1.05 (0.92-1.2)** | **1.08 (1.03-1.13)/0.003** |
| **Net Outcome**** | **719/4920** | **5.62** | **Ref** | **590/4920** | **4.42** | **0.78 (0.69-0.88)** | **651/4920** | **4.86** | **0.84 (0.75-0.94)** | **989/4920** | **7.84** | **1.35 (1.21-1.5)** | **1.19 (1.15-1.24)/<0.001** |

***Model adjusted for baseline SBP, congestive heart failure, hypertension, age, diabetes mellitus, stroke/transient ischemic attack, vascular disease including either myocardial infarction or peripheral arterial disease, and sex).**

**** Net outcome: Stroke, major bleeding, cardiovascular death**

**CV – cardiovascular, HF – heart failure, HHF – hospitalized for heart failure, ICH – intracranial haemorrhage, SBP-systolic blood pressure.**
